# A Cross-Sectional Study on the Association between Body Mass Index and Frailty According to Sex in Elderly Patients with Disabilities from an Elderly Day-Care Center

**DOI:** 10.3390/geriatrics7010007

**Published:** 2021-12-29

**Authors:** Tsuyoshi Asai, Masanori Wakida, Ryo Kubota, Yoshihiro Fukumoto, Haruhiko Sato, Jiro Nakano, Kimitaka Hase

**Affiliations:** 1 Faculty of Rehabilitation, Kansai Medical University, Hirakata, Osaka 573-1136, Japan; wakidam@makino.kmu.ac.jp (M.W.); fukumoty@hirakata.kmu.ac.jp (Y.F.); satohar@makino.kmu.ac.jp (H.S.); nakanoj@hirakata.kmu.ac.jp (J.N.); 2 KMU Day-Care Center Kori, Kansai Medical University Kori Hospital, Neyagawa, Osaka 572-8551, Japan; rkubota0107@msn.com; 3 Department of Physical Medicine and Rehabilitation, Kansai Medical University, Hirakata, Osaka 573-1010, Japan; hasekim@hirakata.kmu.ac.jp

**Keywords:** body mass index, elderly, frailty, physically disabled, sex

## Abstract

The association between body mass index (BMI) and frailty in elderly patients with disabilities is unclear. We aimed to investigate the association between BMI and frailty in the elderly with disabilities according to sex. This cross-sectional study included 280 elderly patients with disabilities from an elderly daycare center. BMI classification for the Asian population was used to categorize the patients into four groups: underweight, normal, overweight, and obese. Frailty score was based on the phenotypic definition of frailty and consisted of five criteria derived from the revised Japanese version of the Cardiovascular Health Study. Those who had three or more criteria were considered frail. Logistic regression models were constructed to investigate the associations between frailty and BMI in each group (males and females). In females, being underweight was significantly associated with frailty after adjusting for confounders (age and Mini-Mental State Examination score); after adding medical history as a confounder, the aforementioned association was not significant. In males, BMI was not significantly associated with frailty. The association between BMI and frailty differed according to sex among the elderly with disabilities. This finding provides important information regarding frailty risk to workers in daycare facilities.

## 1. Introduction

Frailty is a conceptualized, comprehensive, physiological change among the elderly [1,2]. It is defined as “a clinically recognizable state of increased vulnerability resulting from aging-associated decline in reserve and function across multiple physiologic systems, such that the ability to cope with daily or acute stressors is compromised” [1]. Many studies have shown that frailty is strongly associated with health-related outcomes, including mortality, falls, decline in activities of daily living, hospitalization, and health care costs [1,2,3,4,5,6,7,8].

According to community-based research, approximately 10% of the elderly population is estimated to have frailty [9]. Additionally, most elderly people with disabilities who utilized long-term care services were considered frail [9,10,11]. Therefore, this is a serious concern in elderly daycare centers, wherein elderly with disabilities are provided with various services under long-term care, such as rehabilitation. A better understanding of frailty is required to prevent nonfrail elderly individuals from developing such.

Body mass index (BMI) is easily measurable and provides informative data for the elderly. Previous studies investigating the association between BMI and frailty have been conducted among independent community dwellers [12,13,14]. BMI has been reported as one of the factors associated with frailty, and the association has been observed to be U-shaped [12,13,14]. However, results from previous studies cannot be applied to elderly patients with disabilities due to lifestyle differences. Another study from China included people who had similar demographics to those who had disabilities and reported that among patients who were underweight, those who had low skeletal muscle mass and those with high body fat mass were frail [15]. However, the study did not use the BMI classification for Asian populations as it should be modified according to race [16]. Although people with disabilities are at higher risk of frailty, there is a dearth of knowledge regarding the association between BMI and frailty in this population [9,10,11].

These previous studies investigated the association of BMI among females or used sex as an explanatory factor in multiple analyses, despite including both sexes in the study [12,13,14]. Sex strongly affects BMI; thus, the association of BMI with health outcomes would differ accordingly, as observed in a study showing an association between BMI and mortality [17,18]. Thus, in this study, we investigated the association between BMI and frailty according to sex in a daycare center for elderly people, using the BMI classification for Asian populations. Elderly people with disabilities are susceptible to frailty; therefore, investigating the association between frailty and BMI will provide important information for workers in elderly daycare centers.

## 2. Materials and Methods

### 2.1. Patients

This cross-sectional study initially enrolled 349 elderly patients with disabilities under the long-term care insurance system, who were all intended to begin utilizing one elderly daycare center in Japan from April 2018 to March 2019. Potential participants were convenience sampled. The inclusion criteria were as follows: (i) aged > 65 years and (ii) able to participate in a 10-meter walk test [19]. The exclusion criteria were as follows: (i) inability to answer the questionnaire due to severe cognitive impairment (Mini-Mental State Examination [MMSE] score < 21) and (ii) incomplete data for any of the measurements (e.g., MMSE etc.) [20]. The Research Ethics Committee of Kansai Medical University approved the study (approval number 2018251; approved on 28 May 2019). Informed consent was obtained from all participants prior to participation.

**Figure 1:**
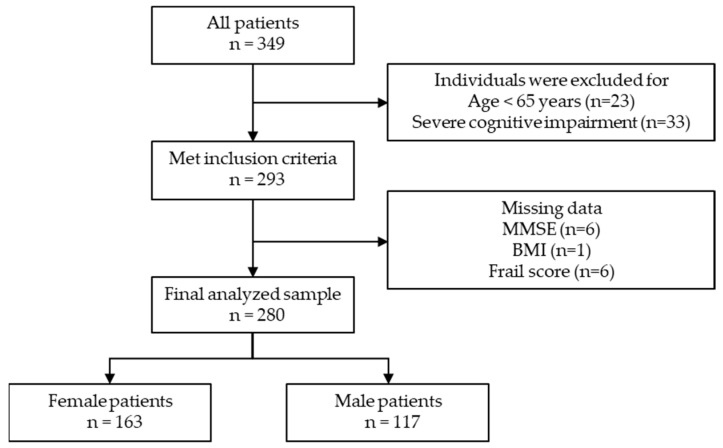
Flow chart of the study sample selection.

Of the 349 participants, 23 were aged < 65 years, 33 had severe cognitive impairment, and 13 had missing data regarding MMSE (n = 6), BMI (n = 1), and frailty (n = 6). The final sample size was 280 (Figure 1, female/male: 163/117).

### 2.2. Data Collection

Demographic data were collected using a self-administered questionnaire with items, including age, sex, and history of fall in the previous year. Fall was defined as “an event that resulted in the participant unintentionally coming to the ground or other lower level” [21]. Data on medical history (musculoskeletal diseases, e.g., spinal and lower limb arthritis, and post-operative pain; neurological diseases, e.g., stroke, heart failure, or cancers) were obtained from the reports of care service managers. Cognitive function was assessed using the MMSE [19]. After obtaining the demographic data, a 10-meter walk test and grip strength test were conducted. In the 10-meter walk test, participants were asked to walk along a 10 m (length) × 2 m (width) smooth horizontal walkway with an extra space of 2 m at both ends of the walkway for acceleration and deceleration [19]. The time taken to complete the 10-m walkway was measured using a digital stopwatch. The 10-m walk test was conducted in accordance with the participants’ comfort and pacing. We confirmed beforehand that the participants wore appropriately sized shoes. Grip strength was measured with a Smedley spring-type dynamometer (GRIP-D; Takei, Niigata, Japan). The measurement was conducted twice [19], and the higher value was used for the assessment of frailty.

### 2.3. BMI Frail Score

A trained physical therapist measured the patients’ height and weight using a measuring tape and digital meter, respectively (AT–WS11, Dretec. Inc., Tokyo, Japan). BMI was calculated as weight in kilograms divided by the height in square meters and was classified according to the criteria proposed for Asian populations (underweight, <18.5 kg/m^2^, normal, 18.5 to 23.0 kg/m^2^, overweight, 23.0 to 27.5 kg/m^2^, obese, ≥27.5 kg/m^2^) [16]. We also categorized the patients according to the WHO classification as a reference (underweight, <18.5 kg/m^2^, normal, 18.5 to 25.0 kg/m^2^, overweight, 25.0 to 30.0 kg/m^2^, obese, ≥30.0 kg/m^2^) [22].

Frailty score was based on the phenotypic definition of frailty, which consists of five criteria derived from the revised Japanese version of the Cardiovascular Health Study: unintentional weight loss in a year (2.0–3.0 kg in the past 6 months), self-reported exhaustion, low physical activity level, slow walking speed (<0.8 m/s), and weakness defined by grip strength (women: < 18 kg, men: < 28 kg) [23]. The first three items were assessed using a self-administered questionnaire, while the last two items were based on the scores of the 10-meter walk and grip strength tests. We counted the applicable items for each subject, and those who met three or more criteria were considered to be frail [23].

### 2.4. Statistical Analysis

Demographic characteristics were compared between both groups (males and females) using the *t*-test for continuous variables and chi-square test for categorical variables. Logistic regression models were constructed to investigate the associations between frailty and BMI in the total study population and in the participants in each group. In Model 1, frailty status was used as the dependent variable, and BMI class was used as the independent variable. In Model 2, sex, age, and MMSE score were included as covariates for all patients, while age and MMSE score were included as covariates for both groups. In Model 3, sex, age, MMSE score, and medical history (musculoskeletal diseases, neurological disease, heart failure, and cancer) were included as covariates for all patients, while age, MMSE score, and medical history were included as covariates for both groups (female and male). Variables of covariates were selected through literature review and clinical experiences in the geriatric field. In all models, odds ratios (ORs) were estimated with reference to the normal BMI group. Statistical analyses were conducted using JMP version software 14.2 (SAS Institute Japan, Tokyo, Japan). Statistical significance was set at a *p*-value of <0.05.

## 3. Results

The demographic data are shown in Table 1. The mean (standard deviation) age of the patients was 79.3 (6.2) years. The mean ages of participants in the female and male groups were 79.4 (5.7) and 79.0 (6.8) years, respectively, with no significant difference between sexes. The number (percentage) of participants in each BMI class (underweight to obese) were 22 (7.9%), 109 (38.9%), 113 (40.4%), and 36 (12.9%), respectively. The number (percentage) of participants in each BMI class (underweight to obese) in the female and male groups were similar and presented as follows: females, 14 (8.6%), 60 (36.8%), 63 (38.7%), and 26 (16.0%) and males, 8 (6.8%), 49 (41.9%), 50 (42.7%), and 10 (8.5%), respectively. Finally, the prevalence of nonfrailty and frailty in all patients was 155 (55.4%) and 125 (44.6%), respectively. The prevalence of nonfrailty and frailty in the female (84 [51.5%] and 79 [48.5%]) and male groups (71 [60.7%] and 46 [39.3%]) was not significantly different.

The results of the logistic regression models are shown in Table 2, Table 3 and Table 4. For all patients in all models, BMI was not significantly associated with frailty. In Models 2 and 3, age and MMSE score were significantly associated with frailty ([Model 2: age: OR, 1.05; 95% confidence interval [95%CI], 1.40–15.94; MMSE: OR, 0.87; 95%CI, 0.96–1.14] [Model 3: age: OR, 1.05; 95%CI: 1.01–1.10; MMSE: OR, 0.89; 95%CI: 0.80–0.98]). In addition, sex was also significantly associated with frailty in Model 3 (sex: OR, 2.00; 95%CI, 1.12–3.61). In the female group, being underweight was significantly associated with frailty in Models 1 and 2; (Model 1: OR, 4.19; 95%CI, 1.06–16.55; Model 2: OR, 4.33; 95%CI, 1.04–18.07). In Model 3, which included the medical history as a covariate, being underweight was not significantly associated with frailty (Model 3: OR, 4.08; 95%CI, 0.93–17.90). In addition, for the female group, age and MMSE were significantly associated in Models 2 and 3 ([Model 2: age: OR, 1.11; 95%CI, 1.04–1.18; MMSE: OR, 0.86; 95%CI, 0.75–0.98] [Model 3: age: OR, 1.10; 95%CI, 1.03–1.18; MMSE: OR, 0.86; 95%CI, 0.75–0.99]). Conversely, in the male group, all variables including the BMI classification were not significantly associated with frailty in all three models (Table 4). The post hoc analysis revealed the average age and standard deviation for frail and nonfrail patients by sex in the underweight group. Among females, the average and standard deviation for frail and nonfrail patients were 80.6 ± 6.7 (n = 11) and 80.3 ± 7.6 (n = 3), respectively. Among males, the average and standard deviation for frail and nonfrail patients were 79.7 ± 2.5 (n = 3) and 79.6 ± 5.0 (n = 5), respectively.

## 4. Discussion

In this study, we investigated the association between frailty and BMI in elderly patients with disabilities from an elderly daycare center. Our hypothesis was that the association between BMI and frailty differs by sex. Frailty was significantly associated with BMI in the female group but not in the male group, while frailty was more prevalent among females in the underweight group than in the normal group. These results indicate that the association between BMI and frailty may differ according to sex and that being underweight is a possible risk factor for frailty in elderly women. However, this association was not significant (*p* = 0.063) after controlling for medical history owing to existing medical conditions. To the best of our knowledge, only two studies have reported an association between BMI and frailty among older women [12,14]. However, they did not provide specific information pertaining to those who were underweight. Blaum et al. showed that frailty scores were high among obese women (BMI > 30 kg/m^2^), but their research did not include underweight women (BMI < 18.5 kg/m^2^) [12]. Sewo et al. showed that frail elderly women had a lower BMI than robust elderly women (Frail vs. Robust, 25 vs. 27.9 kg/m^2^); however, the information regarding underweight older women was unclear [14]. Thus, compared with previous studies, our study provides additional information about the association between frailty and BMI. Considering these results, being underweight is considered a risk factor for elderly women, which warrants attention. Additionally, age is another significant factor for the statistical model. Taken together, older-older female patients who are underweight should be focused upon in clinical settings. Moreover, being overweight rather than obese is acceptable.

In contrast, a risk of frailty based on BMI classification was not observed in the male group, suggesting that a lower BMI may not be a sign of frailty in elderly men. There is a possible explanation for the different associations by sex in this study. One longitudinal multidisciplinary population-based study that investigated the association between change in BMI and survival reported that those with a significant change in BMI (5% BMI gain or loss) showed worse survival rates than those with a stable BMI among youngest-old individuals (age < 79.9 years), but such a difference was not observed among oldest-old individuals (age ≥ 80.0 years) [24]. The mean ages of our study participants in the female and male groups were 79.4 ± 5.7 and 79.0 ± 6.8 years, respectively. Though no significant difference was present, the male participants were substantially older than the female participants, taking the estimated life span for Japanese individuals into consideration (female: 87.5 years, male: 81.4 years) [25]. As a result, the influence of BMI may have become negligibly small, and no association was observed between BMI and frailty in the male group in this study. From the results of the post hoc analysis, sex is considered to be an important factor for frailty; however, this result was derived from a very small sample size. Hence, further study is strongly recommended.

In the present study, we adopted the BMI criteria for the Asian population because the distribution of BMI varies according to race, which can influence the interpretations of results [16,26,27,28]. Consequently, the proportion of those in the normal BMI group was lower, in contrast to those in the overweight and obesity groups, compared to when the WHO classification was used. However, being overweight or obese was not associated with frailty in the logistic regression models, unlike the findings of previous studies from non-Asian countries [12,13]. These results suggest that frailty may not be associated with obesity in people with BMI ranges observed in the present study. In contrast, being underweight was associated with frailty, which was very similar to the result of a previous study conducted in China [15]. This implies that the frailty risk derived from BMI differs according to country.

Notably, our results showed that the MMSE score was an important factor for frailty in both sexes after adjusting for confounders. A systematic review showed that frailty is related to cognitive decline and dementia, which concurs with the results of our study [8]. Cognitive decline and dementia may lead to a reduction in physical activity and changes in eating habits [29]. It is considered that mutual associations among them may lead to frailty. To validate these associations, a well-designed observational study should be conducted, including broader assessments for cognitive function.

There are some limitations to this study. First, this study had a cross-sectional design with convenience sampling; therefore, the causal relationship is unclear and the potential for generalization is limited. Second, the frailty assessment included a question on weight loss. There is a possibility that some patients showed lower BMI values because they had previously lost weight. Third, frailty may have been misclassified due to cognitive decline; some patients were not able to answer the self-administrated questionnaire precisely, and there is a potential inappropriate use of the cut-off value for gait speed for frail classification [30,31]. Such misclassification may have led to a bias in our results. Fourth, the proportions of male participants in the underweight (8) and obesity (10) groups were small; therefore, selection bias may exist. Lastly, there is a lack of important information; other medical histories (e.g., diabetes mellitus (and lifestyle factors, e.g., smoking habits)). These may be potential confounders, which should be included in the statistical models. To address these limitations, a multicenter longitudinal observational study with a larger sample size is warranted.

## 5. Conclusions

The association between BMI and frailty differed by sex in elderly patients with disabilities in elderly daycare centers. Being underweight was associated with frailty in females, and this finding provides important information regarding frailty risk to workers in daycare facilities.

## Figures and Tables

**Table 1:** Demographic characteristics of subjects.

	All Subjects	Female	Male	*p*-Value *
	n = 280	n = 163	n = 117	
Age (y)	79.3 ± 6.2	79.4 ± 5.7	79.0 ± 6.8	0.573
65–74	58 (20.7)	28 (17.2)	30 (25.6)	0.065
75–84	166 (59.3)	106 (65.0)	60 (51.3)	
>84	56 (20)	29 (17.8)	27 (23.1)	
BMI	23.4 ± 3.7	23.7 ± 4.0	23.1 ± 3.2	0.249
BMI groups for Asians, n (%)				
Underweight (<18.5)	22 (7.9)	14 (8.6)	8 (6.8)	0.272
Normal (18.5–23)	109 (38.9)	60 (36.8)	49 (41.9)	
Overweight (23–27.5)	113 (40.4)	63 (38.7)	50 (42.7)	
Obesity (27.5<)	36 (12.9)	26 (16.0)	10 (8.5)	
WHO BMI groups, n (%)				
Underweight (<18.5)	22 (7.9)	14 (8.6)	8 (6.8)	0.361
Normal (18.5–24.9)	173 (61.8)	96 (58.9)	77 (65.8)	
Overweight (25.0–29.9)	71 (25.4)	42 (25.8)	29 (24.8)	
Obesity (30.0<)	14 (5.0)	11 (6.7)	3 (2.6)	
Frail score	2.3 ± 1.2	2.4 ± 1.2	2.2 ± 1.2	0.247
Frail status, n (%)				
Non-frail	155 (55.4)	84 (51.5)	71 (60.7)	0.129
Frail	125 (44.6)	79 (48.5)	46 (39.3)	
Fall history ^a^, n (%)				
Faller	110 (44.2)	63 (42.3)	47 (47.0)	0.462
Non-faller	139 (55.8)	86 (57.7)	53 (53.0)	
MMSE	26.2 ± 2.6	26.3 ± 2.6	26.0 ± 2.7	0.381
TUG (s) ^b^	14.6 ± 7.4	14.5 ± 6.6	14.8 ± 8.5	0.691
Medical History				
Musculoskeletal diseases, n (%)	218 (77.9)	148 (90.8)	70 (42.9)	<0.01
Neurological disease, n (%)	35 (12.5)	9 (5.5)	26 (16.0)	<0.01
Heart failure, n (%)	26 (9.3)	11 (6.7)	15 (9.2)	0.080
Cancer, n (%)	22 (7.9)	4 (2.5)	18 (11.0)	<0.01
mean ± standard deviation				

* Demographic characteristics were compared between sex using *t*-test for continuous variables, and likelihood-test for categorical variables. ^a^ Number of data of fall history in the previous year were 249 (female/male: 149/100). ^b^ Number of data of TUG were 266 (female/male: 154/112). BMI: body mass index, MMSE: mini-mental state examination.

**Table 2:** Odds ratio for frailty in logistic regression model with normal weight as reference in all patients.

All Patients (n = 280)	Model 1 *			Model 2 **			Model 3 ***		
	Adjusted R^2^ = 0.02			Adjusted R^2^ = 0.07			Adjusted R^2^ = 0.09		
	Odds Ratio	95%CI	*p*-Value	Odds Ratio	95%CI	*p*-Value	Odds Ratio	95%CI	*p*-Value
BMI groups ^a^									
Normal	reference			reference			reference		
Underweight	1.92	0.74, 4.94	0.177	1.73	0.65, 4.57	0.270	1.69	0.61, 4.70	0.312
Overweight	0.81	0.47, 1.37	0.434	0.81	0.47, 1.41	0.458	0.82	0.46, 1.44	0.481
Obesity	0.48	0.22, 1.08	0.075	0.49	0.21, 1.13	0.093	0.52	0.22, 1.22	0.131
Female	--------	--------	--------	1.59	0.95, 2.64	0.074	2.00	1.12, 3.61	0.020
Age	--------	--------	--------	1.05	1.40, 15.94	0.011	1.05	1.01, 1.10	0.015
MMSE	--------	--------	--------	0.87	0.96, 1.14	0.005	0.89	0.80, 0.98	0.014
Musculoskeletal diseases	--------	--------	--------	--------	--------	--------	0.97	0.47, 2.01	0.941
Neurological diseases	--------	--------	--------	--------	--------	--------	1.39	0.57, 3.37	0.463
Heart failure	--------	--------	--------	--------	--------	--------	3.85	1.44, 10.27	0.007
Cancer	--------	--------	--------	--------	--------	--------	1.96	0.70, 5.44	0.198

^a^ BMI groups: Patients were categorized into four classes (underweight, normal, overweight, obese). MMSE: mini-mental state examination, 95%CI: 95% confidence interval * Model 1 included BMI class. ** Model 2 included BMI class, sex, age, and MMSE. *** Model 3 included all variables from Model 2 along with locomotive disorder, neurological disease, heart failure, and cancer.

**Table 3:** Odds ratio in logistic regression model for frailty with normal weight as reference in female patients.

Female Patients (n = 163)	Model 1 *			Model 2 **			Model 3 ***		
	Adjusted R^2^ = 0.03			Adjusted R^2^ = 0.11			Adjusted R^2^ = 0.15		
	Odds Ratio	95%CI	*p*-Value	Odds Ratio	95%CI	*p*-Value	Odds Ratio	95%CI	*p*-Value
BMI groups ^a^									
Normal	reference			reference			reference		
Underweight	4.19	1.06, 16.55	0.041	4.33	1.04, 18.07	0.045	4.08	0.93, 17.90	0.063
Overweight	1.11	0.55, 2.25	0.778	1.10	0.52, 2.33	0.796	0.83	0.38, 1.83	0.652
Obesity	0.61	0.23, 1.57	0.302	0.72	0.27, 1.96	0.526	0.66	0.23 1.88	0.440
Age	--------	--------	--------	1.11	1.04, 1.18	0.001	1.10	1.03, 1.18	0.004
MMSE	--------	--------	--------	0.86	0.75, 0.98	0.019	0.86	0.75, 0.99	0.034
Musculoskeletal diseases	--------	--------	--------	--------	--------	--------	0.98	0.23, 4.27	0.979
Neurological diseases	--------	--------	--------	--------	--------	--------	13.62	1.06, 174.20	0.446
Heart failure	--------	--------	--------	--------	--------	--------	2.86	0.55, 14.9	0.210
Cancer	--------	--------	--------	--------	--------	--------	2.21	0.20, 25.06	0.522

^a^ BMI groups: Patients were categorized into four classes (underweight, normal, overweight, obese). MMSE: mini-mental state examination, 95%CI: 95% confidence interval * Model 1 included BMI class. ** Model 2 included BMI class, sex, age, and MMSE. *** Model 3 included all variables from Model 2 along with locomotive disorder, neurological disease, heart failure, and cancer.

**Table 4:** Odds ratio for frailty in logistic regression model with normal weight as reference in male patients.

Male Patients (n = 117)	Model 1 *			Model 2 **			Model 3 ***		
	Adjusted R^2^ = 0.03			Adjusted R^2^ = 0.05			Adjusted R^2^ = 0.09		
	Odds Ratio	95%CI	*p*-Value	Odds Ratio	95%CI	*p*-Value	Odds Ratio	95%CI	*p*-Value
BMI groups ^a^									
Normal	reference			reference			reference		
Underweight	0.63	0.13, 2.91	0.549	0.50	0.10, 2.60	0.430	0.39	0.07, 2.32	0.300
Overweight	0.54	0.24, 1.21	0.132	0.50	0.20, 1.20	0.144	0.56	0.23, 1.35	0.197
Obesity	0.26	0.05, 1.35	0.110	0.20	0.05, 1.33	0.104	0.34	0.06, 1.88	0.215
Age	--------	--------	--------	1.01	0.95, 1.07	0.748	1.01	0.95, 1.07	0.796
MMSE	--------	--------	--------	0.87	0.75, 1.00	0.055	0.87	0.87, 0.76	0.071
Musculoskeletal diseases	--------	--------	--------	--------	--------	--------	1.04	0.43, 2.53	0.932
Neurological disease	--------	--------	--------	--------	--------	--------	0.54	0.18, 1.69	0.292
Heart failure	--------	--------	--------	--------	--------	--------	3.00	0.86, 10.42	0.084
Cancer	--------	--------	--------	--------	--------	--------	2.00	0.61, 6.57	0.249

^a^ BMI groups: Patients were categorized into four classes (underweight, normal, overweight, obese). MMSE: mini-mental state examination, 95%CI: 95% confidence interval * Model 1 included BMI class. ** Model 2 included BMI-class, sex, age, and MMSE. *** Model 3 included all variables from Model 2 along with locomotive disorder, neurological disease, heart failure, and cancer.

## References

[1] Xue Q. (2011). The frailty syndrome: Definition and natural history. Clin. Geriatr. Med..

[2] Fried L.P., Tangen C.M., Walston J., Newman A.B., Hirsch C., Gottdiener J., Seeman T., Tracy R., Kop W.J., Burke G. (2001). Frailty in older adults: Evidence for a phenotype. J. Gerontol. A Biol. Sci. Med. Sci..

[3] Ensrud K.E., Ewing S.K., Taylor B.C., Fink H.A., Cawthon P.M., Stone K.L., Hillier T.A., Cauley J.A., Hochberg M.C., Rodondi N. (2008). Comparison of 2 frailty indexes for prediction of falls, disability, fractures, and death in older women. Arch. Intern. Med..

[4] Clegg A., Young J., Iliffe S., Rikkert M.O., Rockwood K. (2013). Frailty in elderly people. Lancet Lond. Engl..

[5] Han L., Clegg A., Doran T., Fraser L. (2019). The impact of frailty on healthcare resource use: A longitudinal analysis using the clinical practice research datalink in England. Age Ageing.

[6] Bock J.O., König H.H., Brenner H., Haefeli W.E., Quinzler R., Matschinger H., Saum K.U., Schottker B., Heider D. (2016). Associations of frailty with health care costs—Results of the ESTHER cohort study. BMC Health Serv. Res..

[7] Tassiopoulos K., Abdo M., Wu K., Koletar S.L., Palella F.J., Kalayjian R., Taiwo B., Erlandson K.M. (2017). Frailty is strongly associated with increased risk of recurrent falls among older HIV-infected adults: A prospective cohort study. AIDS Lond. Engl..

[8] Hoogendijk E.O., Afilalo J., Ensrud K.E., Kowal P., Onder G., Fried L.P. (2019). Frailty: Implications for clinical practice and public health. Lancet.

[9] Kojima G., Iliffe S., Taniguchi Y., Shimada H., Rakugi H., Walters K. (2017). Prevalence of frailty in Japan: A systematic review and meta-analysis. J. Epidemiol..

[10] Yoshiyuki N., Kono A. (2020). Association between frailty community-dwelling older adults certified as requiring support in the long-term care insurance system and social capital among local neighborhood volunteers. Nihon Koshu Eisei Zasshi.

[11] Boyd C.M., Xue Q.L., Simpson C.F., Guralnik J.M., Fried L.P. (2005). Frailty, hospitalization, and progression of disability in a cohort of disabled older women. Am. J. Med..

[12] Blaum C.S., Xue Q.L., Michelon E., Semba R.D., Fried L.P. (2005). The association between obesity and the frailty syndrome in older women: The Women’s Health and Aging Studies. J. Am. Geriatr. Soc..

[13] Hubbard R.E., Lang I.A., Llewellyn D.J., Rockwood K. (2010). Frailty, body mass index, and abdominal obesity in older people. J. Gerontol. A Biol. Sci. Med. Sci..

[14] Sewo Sampaio P.Y., Sampaio R.A.C., Coelho Júnior H.J., Teixeira L.F.M., Tessutti V.D., Uchida M.C., Arai H. (2016). Differences in lifestyle, physical performance and quality of life between frail and robust Brazilian community-dwelling elderly women. Geriatr. Gerontol. Int..

[15] Xu L., Zhang J., Shen S., Hong X., Zeng X., Yang Y., Liu Z., Chen L., Chen X. (2020). Association between body composition and frailty in elder inpatients. Clin. Interv. Aging.

[16] WHO Expert Consultation (2004). Appropriate body-mass index for Asian populations and its implications for policy and intervention strategies. Lancet.

[17] Di Angelantonio E., Bhupathiraju S., Wormser D., Gao P., Kaptoge S., de Gonzalez A.B., Cairns B., Huxley R., Jackson C., Global BMI Mortality Collaboration (2016). Body-mass index and all-cause mortality: Individual-participant-data meta-analysis of 239 prospective studies in four continents. Lancet Lond. Engl..

[18] Bhaskaran K., dos-Santos-Silva I., Leon D.A., Douglas I.J., Smeeth L. (2018). Association of BMI with overall and cause-specific mortality: A population-based cohort study of 3·6 million adults in the UK. Lancet Diabetes Endocrinol..

[19] Ministry of Education, Culture, Sports, Science and Technology, Japan. https://www.mext.go.jp/a_menu/sports/stamina/03040901.htm.

[20] Folstein M.F., Folstein S.E., McHugh P.R. (1975). “Mini-mental state”. A practical method for grading the cognitive state of patients for the clinician. J. Psychiatr. Res..

[21] Gibson M. (1987). The prevention of falls in later life: A report of the Kellogg International Work Group on the prevention of falls by the elderly. Dan. Med. Bull..

[22] World Health Organization. https://www.who.int/publications/i/item/9789241501491.

[23] Satake S., Shimada H., Yamada M., Kim H., Yoshida H., Gondo Y., Matsubayashi K., Matsushita E., Kuzuya M., Kozaki K. (2017). Prevalence of frailty among community-dwellers and outpatients in Japan as defined by the Japanese version of the Cardiovascular Health Study criteria. Geriatr. Gerontol. Int..

[24] Dahl A.K., Fauth E.B., Ernsth-Bravell M., Hassing L.B., Ram N., Gerstof D. (2013). Body mass index, change in body mass index, and survival in old and very old persons. J. Am. Geriatr. Soc..

[25] Ministry of Health, Labour and Welfare, Japan. https://www.mhlw.go.jp/toukei/saikin/hw/life/life19/.

[26] Stevens J. (2003). Ethnic-specific revisions of body mass index cutoffs to define overweight and obesity in Asians are not warranted. Int. J. Obes..

[27] He W., Li Q., Yang M., Jiao J., Ma X., Zhou Y., Song A., Heymsfield S.B., Zhang S., Zhu S. (2015). Lower BMI cutoffs to define overweight and obesity in China. Obesity.

[28] Caleyachetty R., Barber T.M., Mohammed N.I., Cappuccio F.P., Hardy R., Mathur R., Banerjee A., Gill P. (2021). Ethnicity-specific BMI cutoffs for obesity based on type 2 diabetes risk in England: A population-based cohort study. Lancet Diabetes Endocrinol..

[29] Arvanitakis Z., Shah R.C., Bennett D.A. (2019). Diagnosis and management of dementia: Review. JAMA.

[30] Jung H.W., Jang I.Y., Lee C.K., Yu S.S., Hwang J.K., Jeon C., Lee Y.S., Lee E. (2018). Usual gait speed is associated with frailty status, institutionalization, and mortality in community-dwelling rural older adults: A longitudinal analysis of the Aging Study of Pyeongchang Rural Area. Clin. Interv. Aging.

[31] Clegg A., Rogers L., Young J. (2015). Diagnostic test accuracy of simple instruments for identifying frailty in community-dwelling older people: A systematic review. Age Ageing.

